# Relationship between Mindful Eating and Academic Burnout Syndrome in Medical Undergraduate Students: A Cross-Sectional Study

**DOI:** 10.2174/0117450179460389260309082837

**Published:** 2026-03-16

**Authors:** Nikita Widya Permata Sari, Bayu Satria Wiratama, Fitrina M. Kusumaningrum, Emy Huriyati

**Affiliations:** 1 Department of Health Behavior, Environment, and Social Medicine, Faculty of Medicine, Public Health, and Nursing, Gadjah Mada University, Yogyakarta, Indonesia; 2 Department of Biostatistics, Epidemiology and Population Health, Faculty of Medicine, Public Health, and Nursing, Gadjah Mada University, Yogyakarta, Indonesia; 3 Department of Health Nutrition, Faculty of Medicine, Public Health, and Nursing, Gadjah Mada University, Yogyakarta, Indonesia

**Keywords:** Burnout syndrome, Academic, Undergraduate students, Mindful eating, Distraction, Physical activity

## Abstract

**Introduction/Background:**

Burnout syndrome is an important issue in academic settings. Mindful eating offers a promising approach for intervention strategies aimed at preventing burnout. Exploring the relationship between mindful eating and burnout syndrome may provide a foundation for developing effective interventions to reduce burnout.

**Method:**

This cross-sectional study included 472 undergraduate students from the Faculty of Medicine, Public Health, and Nursing, Gadjah Mada University, representing both medical and non-medical study programs. The Maslach Burnout Inventory (MBI) was used to assess burnout syndrome, while the Mindful Eating Questionnaire (MEQ) measured the dimensions of mindful eating. Simple logistic and ordinal logistic regression analyses were applied to the data.

**Results:**

More than 25% of respondents experienced burnout syndrome, characterized by a moderate degree of emotional exhaustion and a lack of personal accomplishment. Among medical students, distraction was the dominant mindful eating dimension, whereas awareness was predominant among non-medical students. After adjusting for confounders, distraction (aOR = 0.45, *p* < 0.05), external cues (aOR = 2.23, *p* < 0.05), and moderate physical activity (aOR = 2.02, *p* < 0.05) were significantly associated with burnout syndrome. Problem-focused coping acted as a protective factor against emotional exhaustion (aOR = 0.32, *p* < 0.05).

**Discussion:**

These findings contribute to understanding the prevalence and determinants of burnout syndrome among medical and non-medical undergraduate students, with potential implications for other faculties.

**Conclusion:**

Distraction, external cues, problem-focused coping, and moderate physical activity should be considered in faculty program development to help reduce burnout syndrome.

## 1 INTRODUCTION

Burnout syndrome is a state of psychological exhaustion that warrants serious attention because of its broad effects on health and productivity. It is characte-rized by three core dimensions-emotional exhaustion, depersonalization, and reduced personal accomplishment-that manifest chronically in affected individuals [1, 2]. Individuals experiencing burnout commonly exhibit social withdrawal, decreased performance, and depressive mood [2]. Persistent burnout is associated with adverse physical and psychological outcomes, including increased cardiovascular risk, metabolic disorders, and premature mortality [3]. In addition, burnout has been linked to anxiety and depression and has been identified as a moderating factor that weakens the association between work-related quality of life and productivity [4, 5].

Burnout frequently arises in high-pressure environments, particularly the workplace, where excessive demands, heavy workload, and poor relationships are predictors [6, 7]. Similar associations have been observed among academic staff, in which inadequate salaries-additional employment, and stressful conditions exacerbate burnout [8]. Beyond the workplace, burnout is also prevalent in academic settings, particularly among university students. Academic burnout is defined as fatigue, loss of motivation, and reduced engagement in learning activities, ultimately impairing academic performance [9, 10].

Evidence shows that medical students experience particularly high levels of academic burnout. A multi-country study revealed that more than one-third of medical students were affected. In contrast, another large-scale study involving 11,200 students across medical and nursing fields found high prevalence in emotional exhaustion (55.4%), cynicism (31.6%), and low academic efficacy (30.9%) [11, 12]. In Indonesia, the prevalence is equally concerning; research at Gadjah Mada University found that undergraduate students in the Medicine, Nursing, and Nutrition programs showed 100% high-level depersonalization and 92.9% high-level emotional exhaustion [13].

Mindfulness-based approaches have been proposed as promising interventions for reducing burnout. Mindfulness is defined as maintaining non-judgmental awareness of the present moment [14]. Several studies demonstrate its effectiveness in alleviating burnout [15, 16]. One specific application is mindful eating, which involves awareness of thoughts, bodily sensations, emotions, and behaviors during eating [17]. Research has shown that mindful eating correlates negatively with depression among university students [18], positioning it as a potentially valuable strategy to address academic burnout. Recent evidence further supports the broader mental health benefits of mindful eating. A recent study found that it reduced depressive symptoms among older adults [19] and demonstrated its association with resilience in working populations [20]. Since resilience is a critical factor in coping with stress and adversity, these findings reinforce the relevance of mindful eating as a protective mechanism against burnout.

Based on the issues and previous studies, academic burnout syndrome is not a trivial problem and warrants attention, as it can lead to serious health consequences. The potential of mindful eating as a preventive strategy against academic burnout among students deserves serious consideration. Exploring the relationship between mindful eating and academic burnout represents an important step toward generating new insights and recommendations for appropriate interventions to mitigate academic burnout syndrome. Therefore, this study aims to investigate the relationship between mindful eating and academic burnout syndrome among medical undergraduate students.

## 2 METHODS

This research was conducted using a cross-sectional study design from March to April 2025, following ethical approval issued in February 2025. The study population comprised all active undergraduate students of the Faculty of Medicine, Public Health, and Nursing at Gadjah Mada University in Yogyakarta, Indonesia. This population was considered because health students have a higher academic burden, which may lead to burnout [15, 21].

The research sample comprised undergraduate students from the 2021 to 2024 intakes at the Faculty of Medicine, Public Health and Nursing, Gadjah Mada University. The respondent’s inclusion criteria are active undergraduate students from the 2021 to 2024 intake in medical (regular and international) and non-medical study programs (nursing and health nutrition). This study program was divided to enable comparison and identify significant differences in knowledge and behavior resulting from distinct curriculum focuses. The respondent was also willing to participate by completing the informed consent. Respondents who had ever been diagnosed with a serious psychological problem other than burnout syndrome were excluded. The serious psychological problem other than burnout syndrome refers to a clinically diagnosed mental health condition undergoing active treatment. The exclusion was based on a self-report screening question regarding an active clinical diagnosis and ongoing treatment for a major psychiatric condition (*e.g.*, Major Depression, Bipolar Disorder, Schizophrenia). This procedure was adopted to minimize confounding from more severe clinical conditions requiring substantial medical intervention.

Sample size was calculated with the Kish-Leslie formula. This method was previously applied in burnout syndrome research among medical students in Uganda [22]. Using a 95% confidence interval (Z = 1.960), a precision estimate (d = 5%), and an estimated high degree of burnout prevalence (*p* = 63%) from a study in Malaysia [23], the preliminary sample size was 358. The final required sample size was adjusted to 473 students after incorporating a design effect and accounting for non-response data.

This study employed univariate, bivariate, and multivariate analyses to investigate the relationship between mindful eating and burnout syndrome among undergraduates. Univariate analysis used descriptive statistics to determine demographic and academic characteristics. Demographic characteristics are age and gender, while academic characteristics are academic year and credits. Bivariate analysis used simple logistic regression to examine the relationship between variables. Multivariate analysis used ordinal logistic regression to control for confounding variables and to determine the relationship between mindful eating and burnout syndrome at the 95% confidence level. In addition, T-Tests, Chi-Square tests, and Kruskal-Wallis tests were conducted to determine differences in distribution, median, or average between study program groups. The Maslach Burnout Inventory (MBI) was used to assess burnout syndrome, while the Mindful Eating Questionnaire (MEQ) was used to measure mindful eating dimensions. Confounding variables are coping type, physical activity, gender, age, study program, academic year, and credits. Gender, age, study program, academic year, and credits were measured using an ordinal scale. Coping type was measured using the Coping Orientation to Problems Experienced (Brief-COPE) questionnaire, which includes problem-focused, emotion-focused, and avoidant coping [24]. These dimensions were measured using a ratio scale by averaging scores across a specified number of questions. Physical activity was measured using the Global Physical Activity Questionnaire (GPAQ), which categorizes activity as low, moderate, high, or sedentary. These physical activity dimensions were measured using an ordinal scale, except for the sedentary dimension, which was measured by the mean on a ratio scale [25]. All questionnaires were translated into Indonesian and tested for validity and reliability.

The Maslach Burnout Inventory (MBI) was used to assess burnout syndrome, which is divided into three dimensions: emotional exhaustion, depersonalization, and feelings of personal accomplishment. Each dimension is categorized into three levels based on an established cutoff [1]. The level of burnout syndrome was measured using an ordinal scale: low (score 0–1.49), moderate (score 1.5–3.49), and high (score 3.5–6). The burnout syndrome status score was obtained by summing the following: (0.4 x average emotional exhaustion score) + (0.3 x average depersonalization score) + (0.3 x average feeling of lack of personal accomplishment score) [1, 26]. The Mindful Eating Questionnaire (MEQ) was used to measure mindful eating dimensions. Mindful eating is divided into dimensions of awareness, distraction, disinhibition, emoti-onal response, and external cues. These dimensions were measured using a ratio scale by averaging scores across a specified number of questions. A higher average score generally corresponds to a lower body mass index. A higher average dimension score indicates better mindful eating [27].

## 3 RESULTS

**Fig. (1):**
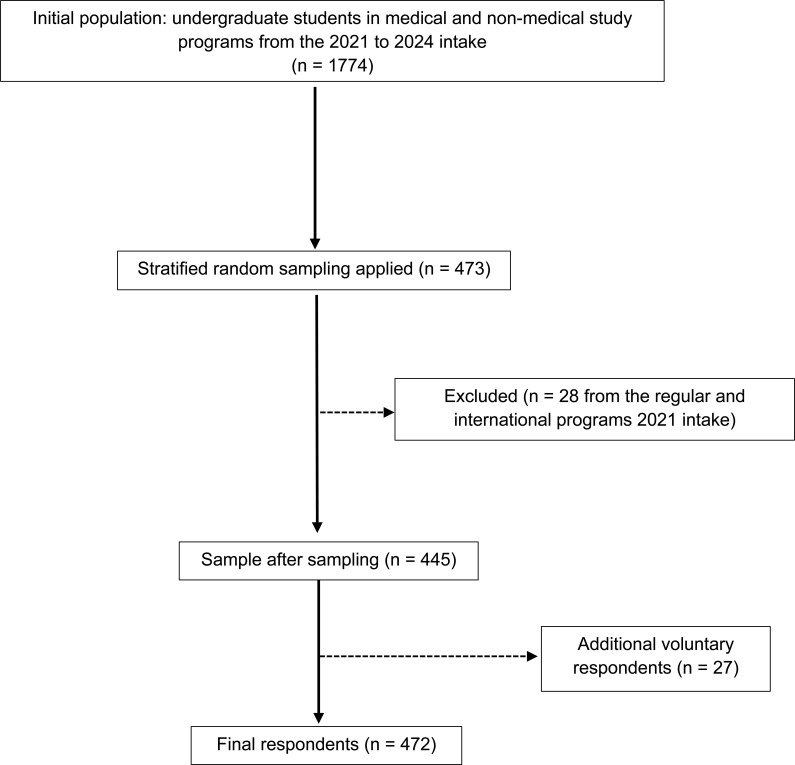
Flow diagram for respondents at each stage of the study.

Respondents in this study were 445 undergraduate students from the 2021 to 2024 intake. The sample selection process involved stratified sampling to ensure proportional representation across academic years and study programs from 1774 undergraduate students, followed by convenience sampling for voluntary participation through an online self-administered questionnaire. If the minimum sample size is not met or if any questionnaires are incomplete (missing data), the respondents will be replaced with other respondents who meet the criteria and complete the questionnaire. However, 28 students from the 2021 intake of the regular and international medicine programs were excluded as they had entered the medical internship period. In the end, the total number of respondents was 472. Respondents were divided into medical students (Regular and IUP) and non-medical students (Nursing and Health Nutrition). Regular Medical Students from the 2021 intake were excluded because their activities were predominantly clinical and differed from those of undergraduate students (Fig **1**). Respondent characteristics are shown in Table **1**.

Respondents to the burnout syndrome questionnaire are divided into three categories, as shown in Table **2**.

Dimensions of burnout syndrome. Respondents reported low-grade emotional exhaustion at 61.7% in the medical study program and 69.4% in the non-medical study program. The second-largest dominance was the burnout syndrome dimension, with respondents reporting a decrease in the sense of low-grade self-achievement, with percentages above 40% for both study program groups. Although low-grade, more than 25% of students experience moderate burnout syndrome in each dimension. The difference test is used to assess differences in the percentage distributions of the burnout syndrome dimensions. The choice of tests is adjusted based on the ordinal scale using Pearson's Chi-Square test. The test results show statistical significance for the feeling of a lack of personal accomplishment dimension among students in non-medical study programs (*p*-value < 0.05). Mindful image eating is divided into five dimensions, while coping types are described in three dimensions. Mindful Eating and Coping Types: Respondents are shown in Table **3**.

The highest average score for each dimension across the Medical and Non-Medical study programs indicated mindful image-eating and coping among respondents. The results of the analysis of the average and standard deviation of the mindful dimension scores, eating, and coping types show that all students in both study programs have a relatively similar average distribution of questionnaire scores. In the medical study program, the average score for the mindful dimension 'eating' is distraction; in the non-medical program, it is awareness. Meanwhile, the highest average score for the coping dimension is problem-focused coping in both study programs. The difference test is used to determine whether the distributions of respondent scores differ between study programs. The T-test is used because both dimensions of the variables are normally distributed and ratio-scaled. The difference test between study program groups did not show any significant differences. The description of respondents' physical activity is divided into low, moderate, high, and sedentary levels. The description of respondents' physical activity levels is shown in Table **4**.

In the medical study program, students' physical activity is dominated by moderate activity, with a 50% share. Meanwhile, the non-medical study program is characterized by low physical activity, with 51% of participants reporting low physical activity. Sedentary activity between study programs has a tendency for the same time of around 7 hours. The difference test is used to determine whether the percentage distributions of respondents' physical activity levels and the average hours of sedentary activity differ between study programs. The use of the difference test is adjusted to the variable scale. Pearson's Chi-Square Test was used for low-to-high activity categories, while the T-Test was used for sedentary activities. The difference test showed statistically significant values for the physical activity category (*p*-value < 0.001). The difference test also showed that medical students were more physically active than non-medical students.

Mindful relationships, eating, and burnout syndrome were analyzed using a simple logistic regression test. The variables tested were the mindful dimension of eating and burnout syndrome, and the confounding variables (sociodemographic, coping type, academic, and physical activity) on burnout syndrome. The results of the simple logistic regression analysis of mindful eating and burnout syndrome are shown in Table **5**.

Dimensions of awareness, distraction, and external cause are statistically significantly related to burnout syndrome. Based on the OR value, an increase in the awareness dimension score is associated with a 2.14-fold greater likelihood of experiencing burnout syndrome. Increased external dimension scores are associated with a 2.56 times greater chance of experiencing burnout syndrome. Conversely, an increase in the distraction dimension score is related to the opportunity for a reduced risk of burnout syndrome by 62%.

The results of the simple logistic regression analysis of mindful eating and confounding variables are shown in Table **6**. Coping type dimensions like emotion-focused coping and avoidant coping, as well as moderate and sedentary activity, are statistically significantly related to burnout syndrome. Emotion-focused coping and avoidant coping have a direct relationship with the occurrence of burnout syndrome. Increased emotion scores and focused coping were associated with a 2.73 times greater chance of experiencing burnout syndrome. Avoidant coping is associated with a higher chance of experiencing burnout syndrome, namely 6.87 times. The results of the physical activity variable also showed a direct relationship with the incidence of burnout syndrome. An increase in the duration of moderate physical activity was associated with a 2.09-fold increase in the likelihood of experiencing burnout syndrome. Compared to low physical activity. Increased sedentary activity increases the chance of burnout syndrome by 1.07 times compared to low physical activity.

The relationship of all variables, including consideration of confounding variables (mindful dimension), eating, sociodemographic, coping type, academic, and physical activity. The symptoms of burnout were analyzed using ordinal logistic regression. The results of the ordinal logistic regression are shown in Table **7**.

The results show that the statistically significant related variables include mindful eating and physical activity. Based on the aOR value, an increase in the distraction dimension score is associated with a decreased risk of burnout syndrome, by 55%. An increase in external dimension score is associated with a 2.23 times greater chance of experiencing burnout syndrome. Meanwhile, increasing the duration of moderate physical activity is associated with a 2.02-fold greater risk of burnout syndrome. compared to low physical activity. Furthermore, the logistic regression results also examined the relationships between mindfulness, eating, and coping types and burnout syndrome per dimension. The results of the mindful logistic regression test, with eating and coping types as predictors, for burnout syndrome per dimension, are shown in Table **8**.

Based on multiple logistic regression, several dimensions of mindful eating and coping styles showed statistically significant associations with burnout dimensions (Table **8**). For emotional exhaustion, distraction during eating and problem-focused coping functioned as protective factors, each associated with approximately a 60% reduction in the odds of emotional exhaustion (aOR = 0.57 and 0.32, respectively). In contrast, avoidant coping was associated with nearly ninefold higher odds of emotional exhaustion (aOR = 8.94). For depersonalization, distraction during eating and problem-focused coping were again protective, each associated with roughly a 50–60% reduction in the odds of depersonalization (aOR = 0.56 and 0.40, respectively), while emotional response, eating, and avoidant coping were associated with higher odds of depersonalization (aOR = 1.70 and 17.31, respectively). Regarding feeling a lack of personal accomplishment, awareness during eating, emotional response eating, problem-focused coping, emotion-focused coping, and avoidant coping were all significantly associated with this dimension. Awareness, problem-focused coping, and emotion-focused coping were associated with higher odds of feeling a lack of personal accomplishment (aOR = 1.72, 2.49, and 3.28, respectively). In contrast, emotional response eating and avoidant coping acted as protective factors, reducing the odds of low personal accomplishment by about 47% and 73%, respectively (aOR = 0.53 and 0.27).

## 4 DISCUSSION

This study found that the mindful eating domains of awareness, distraction, and external cues were significantly associated with burnout syndrome in bivariate analyses. Awareness and external cues were positively correlated with burnout, whereas distraction appeared to be protective. Within the mindful eating framework, awareness reflects attention to the sensory qualities of food (*e.g.*, taste, texture, smell), distraction denotes reduced attentional focus while eating, and external cues capture eating in response to environmental or situational stimuli [27, 28]. Theoretically, awareness may be interpreted through an existential perspective in which self-awareness is central to psychological well-being; diminished existential awareness has been proposed to increase vulnerability to burnout [29, 30]. In contrast, distraction and external cues may be understood within the Job Demands–Resources (JD–R) model, insofar as they reflect external demands or contextual pressures that disrupt mindful eating. Distraction (*e.g.*, using digital devices while eating) can impair attentional engagement [31], while external cues (*e.g.*, stress, food marketing, or environmental triggers) may elicit reactive eating [32]. From a mindfulness standpoint, such cues are ideally met with non-reactivity rather than automatic behavioural responses.

Although prior studies have not explicitly examined links between distraction or external cues and burnout, it is plausible that both undermine mindfulness and contribute to cumulative psychological strain. Mindfulness has been consistently associated with lower levels of psychological distress, and sustained distress may increase risk for burnout and depressive symptoms [33]. Notably, this study yielded an unexpected pattern: awareness was positively associated with burnout, suggesting that higher awareness scores may coincide with greater burnout risk. This finding contrasts with prior evidence indicating that awareness is inversely related to perceived stress among university students in the United States, implying that greater mindfulness may reduce stress [34].

This study also indicated that external cues increased the likelihood of burnout, whereas distraction was associated with lower burnout. Prior research has more often focused on distraction as a potential coping response, particularly in relation to depressive symptoms. For example, a study at the University of Brighton (United Kingdom) reported that distraction and emotional responding during eating were associated with lower depression and binge eating among health students [18]. Other studies similarly suggest that listening to music while eating may help regulate negative affect; however, maintaining an appropriate balance is important to minimize the risk of overeating [35, 36].

Discrepancies with earlier findings may reflect differences in mindful eating profiles across student groups. In this sample, medical students tended to score higher on distraction, whereas non-medical students (nursing and nutrition) demonstrated greater awareness. Mindful eating styles are complex and may differentially shape behavioral and psychological responses; however, much of the existing literature has emphasized dietary intake and body weight outcomes rather than mental health dimensions [17, 37]. Several factors that may have confounded observed associations-including coping styles (emotion-focused and avoidant) and moderate or sedentary physical activity-were also significantly related to burnout and may have influenced the primary results. In addition, the self-administered survey format, distributed without researcher supervision, may have introduced response bias [38, 39]. Other unmeasured factors, including cultural and demographic characteristics, may further shape the magnitude and direction of associations.

In multivariable ordinal logistic regression adjusting for confounders, distraction remained protective, and external cues remained positively associated with burnout. Interestingly, moderate physical activity was also associated with higher burnout, diverging from evidence that physical activity is generally protective for mental health [40]. This discrepancy may reflect unexamined mediators or the possibility that students perceive physical activity as burdensome or time-consuming rather than restorative [41].

Dimension-specific analyses further indicated that distraction, problem-focused coping, and avoidant coping were significant predictors of emotional exhaustion. Avoidant coping, in particular, increased the odds of both emotional exhaustion and depersonalization [42, 43]. By contrast, problem-focused coping and aspects of mindful eating were associated with lower symptom levels, highlighting their potential protective roles.

For reduced personal accomplishment, awareness, emotional response, and multiple coping strategies were significantly associated. Emotion-focused coping showed the strongest association (OR = 3.28), followed by problem-focused coping (OR = 2.49), whereas avoidant coping and mindful eating appeared protective. Prior work suggests that emotion-focused coping may mediate the relationship between stress and adverse psychological outcomes, although causal inference remains limited because much of the literature relies on cross-sectional designs [44, 45].

Overall, most students reported low burnout; however, more than 25% experienced moderate burnout in each dimension. Previous research has identified emotional exhaustion as a prominent component of medical student burnout globally and has reported higher prevalence among female students [46]. Studies in other settings have similarly documented substantial emotional exhaustion and academic inefficacy among medical students, including in Iran [47]. Collectively, these findings underscore the importance of addressing burnout within higher education.

These results suggest that program refinement within Health Promoting University (HPU) strategies at Universitas Gadjah Mada should consider the roles of distraction, external cues, coping styles (particularly problem-focused coping), and students’ lived experiences of physical activity. The findings indicate that students may rely on maladaptive coping approaches, including avoidance and emotion-focused coping. Programs that integrate contemplative practices with emotional regulation training-such as the Be REAL program-may offer a relevant model for strengthening mental health initiatives [48]. Within HPU programming, evaluation efforts could prioritize interventions that address external cues and cultivate adaptive forms of attentional engagement, while physical activity initiatives should assess students’ perceived benefits and barriers. Evidence suggests that low-to-moderate intensity physical activity can support mental health outcomes [49, 50]; accordingly, HPU initiatives may emphasize appropriate exercise type, timing, and intensity. Strong institutional commitment, including formal evaluation mandates, may further strengthen program implementation.

In summary, these findings contribute to understanding the prevalence and correlates of burnout among undergraduate students at the Faculty of Medicine, Universitas Gadjah Mada, and may have relevance for other faculties. Future research should examine potential causal pathways linking mindful eating and burnout and incorporate qualitative approaches to capture students’ lived experiences. Longitudinal studies may also help inform campus health promotion interventions that integrate adaptive coping, context-sensitive attention to eating, and supportive physical activity to improve student mental well-being.

## CONCLUSION

Mindful eating (distraction and external cues), problem focused-coping, and moderate physical activity were statistically significantly related to burnout syndrome after controlling for confounding variables. The faculty or university may consider these related findings to gradually evaluate the HPU program, particularly regarding mental health initiatives within the Faculty of Medicine, Public Health, and Nursing, or in other faculties at Gadjah Mada University. Although this cross-sectional study has limitations, such as a lack of supervision during online data collection and self-administered questionnaires, which may cause response bias, this study can be continued with several improvements. Furthermore, this study also has limitations concerning the potential impact of the exclusion criteria on external validity and the generalizability of the findings. We acknowledge that the exclusion criteria may have led to our results underestimating the prevalence of mindful eating or burnout among the population with a higher degree of mental illness. A future study should implement a refined data collection protocol and an enhanced study method. We also suggest that future studies explicitly include and control for mental clinical variables.

## Figures and Tables

**Table 1:** Sociodemographic characteristics of respondents.

Variables	Medical	Non-medical	Total	*p*-value
N	230 (48.7%)	242 (51.3%)	472 (100%)	
Age 1	19 (18-20)	20 (19-21)	20 (19-21)	<0.001*
Gender				
Man	65 (28.3%)	19 (7.9%)	84 (17.8%)	<0.001*
Woman	165 (71.7%)	223 (92.1%)	388 (82.2%)	
Academic year				
2021	0 (0%)	56 (23.1%)	56 (11.9%)	<0.001*
2022	74 (32.2%)	63 (26.0%)	137 (29.0%)	
2023	71 (30.9%)	58 (24.0%)	129 (27.3%)	
2024	85 (37.0%)	65 (26.9%)	150 (31.8%)	
Credit				
24 credits	97 (42.2%)	25 (10.3%)	122 (25.8%)	<0.001*
< 24 credits	133 (57.8%)	217 (89.7%)	350 (74.2%)	

**Note:** N = total respondents; n = number of respondents; ^1^ age using median (Q1-Q3); *Statistically significant difference test

**Table 2:** Description of burnout syndrome of respondents in each study program.

Dimensions	Medical	Non-medical	Total	*p*-value
N	230 (48.7%)	242 (51.3%)	472 (100%)	
Emotional exhaustion (n,%)				
Low degree	142 (61.7%)	168 (69.4%)	310 (65.7%)	0.210
Moderate degree	74 (32.2%)	63 (26.0%)	137 (29%)	
High degree	14 (6.1%)	11 (4.5%)	25 (5.3%)	
Depersonalization (n,%)				
Low degree	80 (34.8%)	96 (39.7%)	176 (37.3%)	0.078
Moderate degree	65 (28.3%)	80 (33.1%)	145 (30.7%)	
High degree	85 (37.0%)	66 (27.3%)	151 (32.0%)	
Feeling Lack of Personal Accomplishment (n,%)				
Low degree	97 (42.2%)	128 (52.9%)	225 (47.7%)	0.018*
Moderate degree	61 (26.5%)	64 (26.4%)	125 (26.5%)	
High degree	72 (31.3%)	50 (20.7%)	122 (25.8%)	

**Note:** N = total respondents; n = number of respondents; *statistically significant difference test

**Table 3:** Respondents' mindful eating and coping types.

Variables	Medical	Non-medical	Total	*p*-value
N	230 (48.7%)	242 (51.3%)	472 (100%)	
Mindful Eating Dimension (mean ± SD)				
Awareness	2.65 ± 0.48	2.70 ± 0.44	2.67 ± 0.46	0.181
Distraction	2.68 ± 0.42	2.69 ± 0.37	2.68 ± 0.39	0.628
Disinhibition	2.55 ± 0.28	2.60 ± 0.30	2.58 ± 0.29	0.057
Emotional response	2.39 ± 0.58	2.42 ± 0.56	2.40 ± 0.57	0.603
External cause	2.33 ± 0.36	2.38 ± 0.36	2.35 ± 0.36	0.127
Coping Type (mean ± SD)				
Problem-focused coping	3.26 ± 0.47	3.22 ± 0.39	3.24 ± 0.43	0.438
Emotion-focused coping	2.98 ± 0.34	2.96 ± 0.32	2.97 ± 0.33	0.581
Avoidant coping	1.94 ± 0.32	1.95 ± 0.31	1.95 ± 0.31	0.698

**Note:** N = total respondents; mean = average; SD = standard deviation

**Table 4:** Overview of respondents' physical activity levels.

Physical Activity	Medical	Non-medical	Total	*p*-value
N	230 (48.7%)	242 (51.3%)	472 (100.0%)	
Physical Activity (n, %)				
Low	60 (26.1%)	123 (51.0%)	183 (38.9%)	<0.001*
Moderate	115 (50.0%)	83 (34.4%)	198 (42.0%)	
High	55 (23.9%)	35 (14.5%)	90 (19.1%)	
Sedentary (mean ± SD)	7.45 ± 3.80	7.59 ± 3.80	7.52 ± 3.80	0.696

**Note:** N = total respondents; n = number of respondents; SD = standard deviation; *test of statistically significant difference

**Table 5:** Result of the simple logistic regression test for mindful eating and burnout syndrome.

Dimensions	OR	*p*-value	95% CI
Awareness	2.14	0.006*	1.24 – 3.70
Distraction	0.38	0.003*	0.20 – 0.71
Disinhibition	1.04	0.924	0.44 – 2.44
Emotional response	1.46	0.086	0.94 – 2.26
External cause	2.56	0.008*	1.28 – 5.14

**Abbreviations:** OR = Odds Ratio; CI = Confidence Interval; * *p*-value < 0.05

**Table 6:** Result of multiple logistic regression test.

Variables	aOR	*p*-value	95% CI
Gender			
Man	Reference		
Woman	0.71	0.287	0.37 – 1.34
Age	0.84	0.087	0.69 – 1.03
Study program			
Non-Medical	Reference		
Medical	1.37	0.208	0.84 – 2.26
Year			
2021	Reference		
2022	1.69	0.206	0.75 – 3.82
2023	2.10	0.082	0.91 – 4.82
2024	1.77	0.165	0.79 – 4.00
Credit			
24 credits	Reference		
< 24 credits	0.75	0.319	0.43 – 1.32
Type coping			
Problem focused coping	1.36	0.297	0.76 – 2.41
Emotion-focused coping	2.73	0.008*	1.30 – 5.74
Avoidant coping	6.87	0.000*	3.12 – 15.12
Physical Activity			
Low	Reference		
Currently	2.09	0.010*	1.19 – 3.69
High	1.71	0.135	0.85 – 3.47
Sedentary	1.07	0.023*	1.01 – 1.15

**Abbreviations:** aOR = adjusted Odds Ratio; CI = Confidence Interval; * *p*-value < 0.05

**Table 7:** Result of multiple ordinal logistic regression test on burnout syndrome.

Variables	aOR	*p*-value	95% CI
Mindful Eating			
Awareness	1.65	0.115	0.88 – 3.09
Distraction	0.45	0.021*	0.23 – 0.88
Disinhibition	0.74	0.531	0.28 – 1.91
Emotional Response	0.85	0.519	0.51 – 1.41
External Cause	2.23	0.049*	1.00 – 4.99
Coping Type			
Problem Focused-Coping	0.71	0.414	0.31 – 1.62
Emotion Focused Coping	2.74	0.067	0.93 – 8.08
Avoidant Coping	7.51	0,000	2.92 – 19.33
Gender			
Man	Reference		
Woman	0.52	0.086	0.24 – 1.10
Age	0.77	0.172	0.53 – 1.12
Credit			
24 credits	Reference		
< 24 credits	0.61	0.155	0.31 – 1.21
Study program			
Non-Medical	Reference		
Medical	0.77	0.420	0.41 – 1.45
Year			
2021	Reference		
2022	1.28	0.629	0.47 – 3.47
2023	1.21	0.754	0.37 – 3.93
2024	0.62	0.515	0.14 – 2.64
Physical Activity			
Low	Reference		
Currently	2.02	0.028*	1.08 – 3.77
High	1.33	0.473	0.61 – 2.86
Sedentary	1.07	0.052	1.00 – 1.15

**Abbreviations:** aOR = Adjusted Odds Ratio ; CI = Confidence Interval ; * *p*-value < 0.05

**Table 8:** Multiple logistic regression test for mindful eating and coping types against burnout syndrome per dimension.

Variables	Emotional Exhaustion	Depersonalization	Feeling Lack of Personal Accomplishment
	aOR (95%CI)	aOR (95%CI)	aOR (95%CI)
Mindful Eating			
Awareness	1.25 (0.76 – 2.06)	1.02 (0.66 – 1.61)	1.72 (1.09 – 2.72)*
Distraction	0.57 (0.33 – 0.98)*	0.56 (0.34 – 0.92)*	0.90 (0.55 – 1.47)
Disinhibition	0.56 (0.25 – 1.26)	0.56 (0.28 – 1.13)	0.92 (0.46 – 1.86)
Emotional Response	0.87 (0.58 – 1.29)	1.70 (1.17 –2.48)*	0.53 (0.37 – 0.77)*
External Cause	1.26 (0.68 – 2.33)	1.56 (0.89 – 2.76)	0.83 (0.48 – 1.46)
Coping type			
Problem Focused-Coping	0.32 (0.16 – 0.62)*	0.40 (0.22 – 0.72)*	2.49 (1.35 – 4.60)*
Emotion Focused Coping	2.56 (0.92 – 5.51)	1.09 (0.50 – 2.35)	3.28 (1.50 – 7.18)*
Avoidant Coping	8.94 (4.21 – 19.02)*	17.31 (8.36 – 35.82)*	0.27 (0.13 – 0.53)*

**Abbreviations:** aOR = Adjusted Odds Ratio; CI = Confidence Interval; * *p*-value < 0.05

## Data Availability

The data and supportive information are available within the article.

## LIST OF ABBREVIATIONS

MBI: Maslach Burnout Inventory
MEQ: Mindful Eating Questionnaire
Brief-COPE: Coping Orientation To Problems Experienced Inventory Questionnaire
GPAQ: Global Physical Activity Questionnaire
HPU: Health Promoting University
CI: Confidence Interval
OR: Odds Ratio
aor: Adjusted Odds Ratio
STROBE: Strengthening The Reporting Of Observational Studies In Epidemiology

## STANDARDS OF REPORTING

STROBE guidelines were followed.
